# Targeting Long Non-Coding RNA TTN-AS1 Suppresses Bladder Cancer Progression

**DOI:** 10.3389/fgene.2021.704712

**Published:** 2021-10-04

**Authors:** Huiyuan Xiao, Wen Huang, Yanlei Li, Rongxin Zhang, Long Yang

**Affiliations:** ^1^ Department of Urology, Tianjin Medical University General Hospital, Tianjin, China; ^2^ Department of Pathology, Tianjin Medical University, Tianjin, China; ^3^ Department of Radiotherapy, Tianjin Medical University General Hospital, Tianjin, China

**Keywords:** long non-coding RNA, bladder cancer, prognosis, oncogene, bioinformactics

## Abstract

**Background:** To explore the biological and clinical effects of titin-antisense RNA1 (TTN-AS1) in bladder cancer (BC) and the association between TTN-AS1 and activating transcription factor 2 (ATF2) in BC.

**Methods:** The Kaplan–Meier method was performed to analyze the association between the expression of TTN-AS1 and prognosis of BC patients from TCGA data set and our institution. Quantitative real-time PCR (RT-PCR) was conducted to explore the expression of TTN-AS1 between the patients who underwent TURBT and Re-TURBT. MTT, colony formation, and tumor formation assays were conducted to evaluate the effect of TTN-AS1 on the ability of proliferation in BC cell lines. Transwell assay was performed to evaluate the effect of TTN-AS1 on the ability of invasion in BC cell lines. Bioinfomatics and immunohistochemical staining was used to identify the relationship between TTN-AS1 and ATF2.

**Results:** The higher expression of TTN-AS1 was related to poorer disease-free survival (DFS) in patients with BC. The expression of TTN-AS1 was higher in BC patients who underwent Re-TURBT compared with BC patients who underwent TURBT. Knocking down TTN-AS1 resulted in inhibiting the ability of proliferation and invasion of BC cells. ATF2 may serve as a downstream target of TTN-AS1 in BC, and the high expression of ATF2 is also related to adverse DFS.

**Conclusion:** Our study reveals that TTN-AS1 serves as an oncogene by activating ATF2 in BC. The findings suggest that TTN-AS1 may act as a novel therapeutic target for patients with BC.

## Introduction

Bladder cancer (BC) is one of the most frequency urinary malignancies in males with high recurrence and poor prognosis (Torre et al., 2015; Siegel et al., 2019). As one of the lethal causes in patients, BC is still a major issue for public health. In patients with non-muscle-invasive BC (NMIBC), the tumor is confined to the mucosa or submucosa of the bladder wall. However, if the tumor invades the muscle layer, it is called muscular invasive BC (MIBC). If the patient with MIBC does not develop distant metastasis or invade adjacent tissues, the bladder and the tumor should be considered to be surgically removed (Hinsenveld et al., 2021). With deepening research, more and more efforts have been made to find effective therapeutic targets against BC improving patient outcomes.

Long noncoding RNA (lncRNA), which is a subtype of RNA defined as transcripts >200 nt without protein-coding ability, is explored often and suggested to have a regulatory role in the development and progression of BC (Lv et al., 2017; Li et al., 2018; Quan et al., 2018; Zhang et al., 2019). A recent study shows that LncRNA NR2F1-AS1 could serve as an independent prognostic factor in BC, and the researchers established a genomic-related nomogram to predict the prognosis of BC (Peng et al., 2021). LncRNA titin-antisense RNA1 (TTN-AS1) transcripts are from the antisense strand of TTN and are suggested to be oncogenic lncRNA in numerous tumors (Chen et al., 2018; Lin et al., 2018; Cui et al., 2019; Dong et al., 2019). However, the clinical relevance and functional role of lncRNA TTN-AS1 has not been investigated in BC. In the present study, we find that highly expressed lncRNA TTN-AS1 is correlated with BC progression. Importantly, lncRNA TTN-AS1 could be upregulated in re-TURBT BC specimens. Then, growth and invasion suppression of BC cells *in vitro* and *in vivo* by targeting lncRNA TTN-AS1 supports its potential therapeutic effects.

Activating transcription factor 2 (ATF2), a member of the AP1 transcription factor family, is demonstrated to be an inducer for BC cells (with functional androgen receptor) proliferation as well as invasion (Inoue et al., 2018). In this study, we explore the relationship between ATF2 and lncRNA TTN-AS1 *in vitro* and *in vivo*. We also demonstrate that ATF2 and lncRNA TTN-AS1 plays a vital role in the progression of BC.

## Materials and Methods

### Ethical Approval for the Study Protocol

This study was approved by the Ethics Committee of Tianjin Medical University General Hospital, Tianjin, China (No. KY2020K09). Written informed consent was obtained from all patients, and the study was conducted in accordance with the Declaration of Helsinki.

### Human Samples

The bladder tissue specimens were surgical specimens from BC patients with complete clinicopathological data. BC tissues that were embedded in paraffin were acquired by transurethral resection of bladder tumor (TURBT) at the Tianjin Medical University General Hospital (*n* = 130). All patients did not receive chemotherapy and radiotherapy before surgery. The fresh paired BC tissues which were recurred (recurrence means that a patient who was diagnosed with BC for the first time has a new tumor in the bladder after undergoing TURBT surgery) were immediately stored in liquid nitrogen after TURBT (*n* = 6).

### Antibodies

The antibodies used in this study are listed as follows: ATF2 (Abcam, ab239361, 1:250 dilution for IHC) and Ki67 (Abcam, ab15580, 1:1,000 dilution for IHC).

### Cell Lines and Cell Culture

Human BC cell line T24 and 5,637 cells were purchased from the American Tissue Culture Collection (ATCC). T24 and 5,637 cells were incubated in RPMI 1640 (Gibco, Waltham, MA United States). All media were supplemented with 10% fetal bovine serum (Gibco, Waltham, MA United States). All experimental cell lines were cultured at 37°C containing 5% CO_2_.

### *In vitro* Transfection

Transfection was performed using cells in the logarithmic growth phase. The cells are seeded into six-well plates at a density of 5 × 105 cells/well. After the cells grow to 60% of the area of each well, transfection is performed. The specific procedure was carried out with Lipofectamine 3,000 according to the manufacturer’s instructions (Invitrogen, Thermo Scientific, MA, United States). The shRNA targeting TTN-AS1 (shTTN-AS1) and the shRNA targeting scramble (shSCR) were obtained from Shanghai GenePharma Co., Ltd (Shanghai, China).

### Polymerase Chain Reaction

Total RNA was extracted using Trizol and reverse transcribed into cDNA. Using the cDNA as a template, PCR was performed to detect the expression of TTN-AS1 using GAPDH as an internal reference. The primer sequences were as follows: TTN-AS1 forward primer, 5′- GCC​AGG​TAG​AGT​TGC​AGG​TT-3′; reverse primer, 5′- GAA​GCT​GCT​GCG​GAT​GAA​TG-3′; GAPDH forward primer, 5′-GAT​TCC​ACC​CAT​GGC​AAA​TT-3′; reverse primer, 5′-TCT​CGC​TCC​TGG​AAG​ATG​GT-3′. Reaction bands were observed using gel electrophoresis.

### MTT Assay

Weigh 0.5 g of MTT [3-(4,5)-dimethylthiahiazo (-z-y1)-3,5-di-phenytetrazoliumromide] order and dissolve it in 100 ml of PBS and pass the solution through a sterile filter membrane with a pore size of 0.22 μm to filter out bacteria in the solution. Add 100 μl of cell culture medium to each well of the 96-well plate and ensure that there are 2 × 103 cells in each well. Place the paved 96-well plate in a constant temperature cell incubator containing 5% CO_2_ at 37°C for culture. After a certain period of time, discard the medium in the well plate, add 100 μl of MTT solution to each experimental well and then place it in a cell culture incubator for 4 h. The supernatant was discarded again, and 150 μl of DMSO was added to each experimental well. Place the 96-well plate on a plate shaker and shake it slowly, and then place the solution to be checked in the microplate reader, measure the optical density at a wavelength of 570 nm, and record the optical density value.

### Colony Formation Assay

When the cells are in the logarithmic growth phase, the BC cells were collected and cultured by a conventional cell culture method. When macroscopic colonies appeared in the Petri dish, the culture was terminated. Discard the supernatant, add 4% paraformaldehyde to fix the cells, and then stain the cells with GIMSA application staining solution. Slowly wash away the staining solution with running water and let the plate dry completely. Finally calculate the number of clones.

### Invasion Transwell Assay

Take 200 µl of the cell suspension and add it to the Transwell chamber to ensure that each well contains 1 × 104 cells. Perform cell culture in a conventional cell culture method. After 48 h, remove the chamber and discard the supernatant. Fix and stain the cells under the Transwell membrane; observe and count under a microscope.

### *In vivo* Experiment

Male nude mice (8 weeks old, *n* = 10) were purchased from Beijing HFK Bioscience Co., Ltd (Beijing, China). Subcutaneous tumor growth assays were performed with T24 cell lines (1 × 106 T24 cells injected in each mouse). The tumor size was measured every 7 days with a Vernier calliper, and the volume change of the tumor was calculated. The tumors were harvested under standard, institutionally approved processes. The mass of each tumor was measured with an electronic balance. Tumor samples were paraffin-fixed and processed for immunohistochemical (IHC) analysis.

### Immunohistochemistry

For IHC staining, paraffin-embedded tissue samples were deparaffinized, rehydrated, and incubated with primary antibodies (rabbit antihuman monoclonal antibody ATF2 antibody) overnight at 4°C. The secondary antibodies (goat antirabbit IgG) were incubated at room temperature for 1 h. The signals were examined under a microscope.

### Statistical Analysis

Statistical significance was determined using two-tailed Student’s *t*-test or ANOVA for functional analysis. Survival curves were plotted using Kaplan–Meier survival plots and a log-rank test was used to test significance. All statistical analyses were performed using SPSS software and GraphPad Prism7. *p* < 0.05 was considered statistically significant. *p* < 0.05 was marked as *, *p* < 0.01 was marked as **, *p* < 0.001 was marked as ***, and no significant difference was expressed as n.s.

## Results

### Highly Expressed lncRNA TTN-AS1 Is Correlated With BC Progression

**FIGURE 1:**
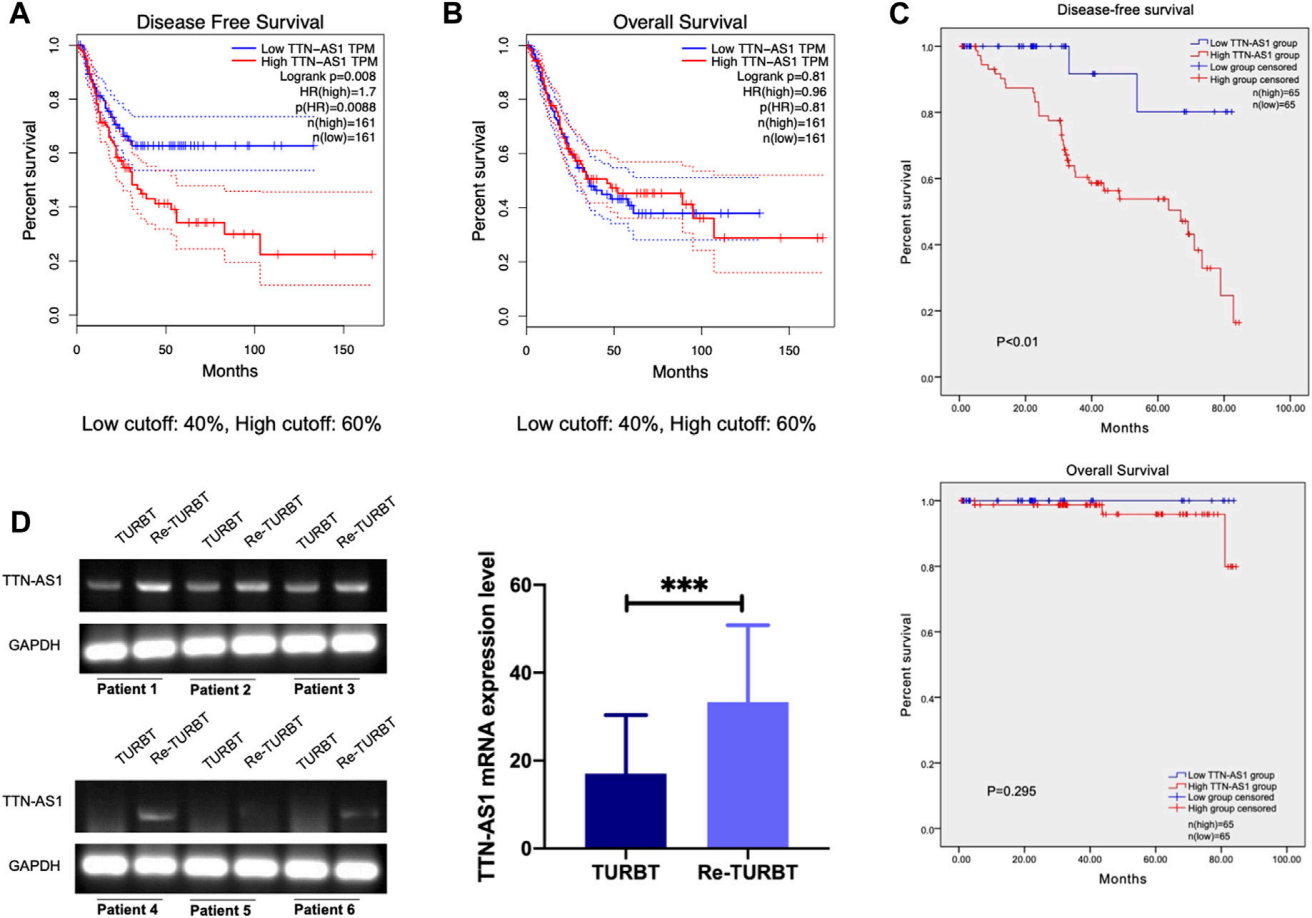
LncRNA TTN-AS1 was upregulated in nonmuscular invasive BC who underwent TURBT and is related with adverse DFS **(A)**. The DFS of BC patients from TCGA database (*n* = 322, *p* = 0.0088) **(B)**. The OS of BC patients from TCGA database (*n* = 322, *p* = 0.81) **(C)** The DFS (*p* < 0.01) and OS (*p* < 0.295) of BC patients from our institution (*n* = 130) **(D)**. qRT-PCR was used to detect the expression level of lncRNA TTN-AS1 at mRNA level in surgical specimens of BC patients who underwent TURBT and Re-TURBT (*** means: *p* < 0.001).

LncRNA TTN-AS1 has been stated and functionally confirmed to be involved in a variety of cancer progressions (Chang et al., 2020; Feng et al., 2020; Lin et al., 2020; Miao et al., 2020; Qi and Li, 2020). Although the role of TTN-AS1 in BC is still poorly characterized. To investigate the clinical relevance of TTN-AS1 expression in BC, we retrieved public TCGA data sets from cBioPortal (Cerami et al., 2012; Gao et al., 2013) and evaluated TTN-AS1 expression in relation to disease-free survival (DFS) data as well as overall survival (OS) data on 322 BC patients. We found that BC patients with TTN-AS1 gene high expression had worse DFS (cutoff high expression: 60% of all patients; cutoff low expression: 40% of all patients) (Figure 1A), and its expression has no prediction of OS (Figure 1B). Except that the DFS of BC patients from our institution was also analyzed. As shown in Figure 1C, the TTN-AS1 high expression group had an adverse DFS, which was coincident with the TCGA database. The association between TTN-AS1 expression and clinical characters in BC patients was also analyzed by using chi-square test. As shown in Table 1, the high TTN-AS1 expression was related with advanced tumor stage (*p* ≤ 0.01), high tumor grade (*p* = 0.02), lymph node metastasis (*p* ≤ 0.01) and vascular invasion (*p* = 0.01).

**TABLE 1:** Clinicopathologic variables and TTN-AS1 expression in 130 bladder cancer patients.

Variables	All *n* = 130	TTN-AS1		*p* value^#^
		Low *n* = 35	High *n* = 95	
Age				
<65	61	16	45	0.87
≥65	69	19	50	
Sex				
Male	54	14	40	0.83
Female	76	21	55	
Tumor stage				
T2	60	25	35	≤0.01
T3/T4	70	10	60	
Tumor grade				
Low	60	22	38	0.02
High	70	13	57	
Lymph node metastasis				
No	50	20	30	≤0.01
Yes	80	15	65	
Vascular invasion				
No	70	25	45	0.01
Yes	60	10	50	

a #*p* value was analyzed by Chi-square test; * indicates *p* < 0.05 with statistical significance; iPSA means initial PSA.

Given the association with BC progression of highly expressed lncRNA TTN-AS1, we performed PCR detection on TURBT and paired re-TURBT specimens (*n* = 6) collected in our hospital. PCR results suggest patients received re-TURBT with recurrence may be correlated with local increased expression of TTN-AS1 (Figure 1D). These results led us to propose the clinical association and oncogenic role of TTN-AS1 in BC.

### Silence of lncRNA TTN-AS1 Suppresses BC Cell Proliferation and Invasion

**FIGURE 2:**
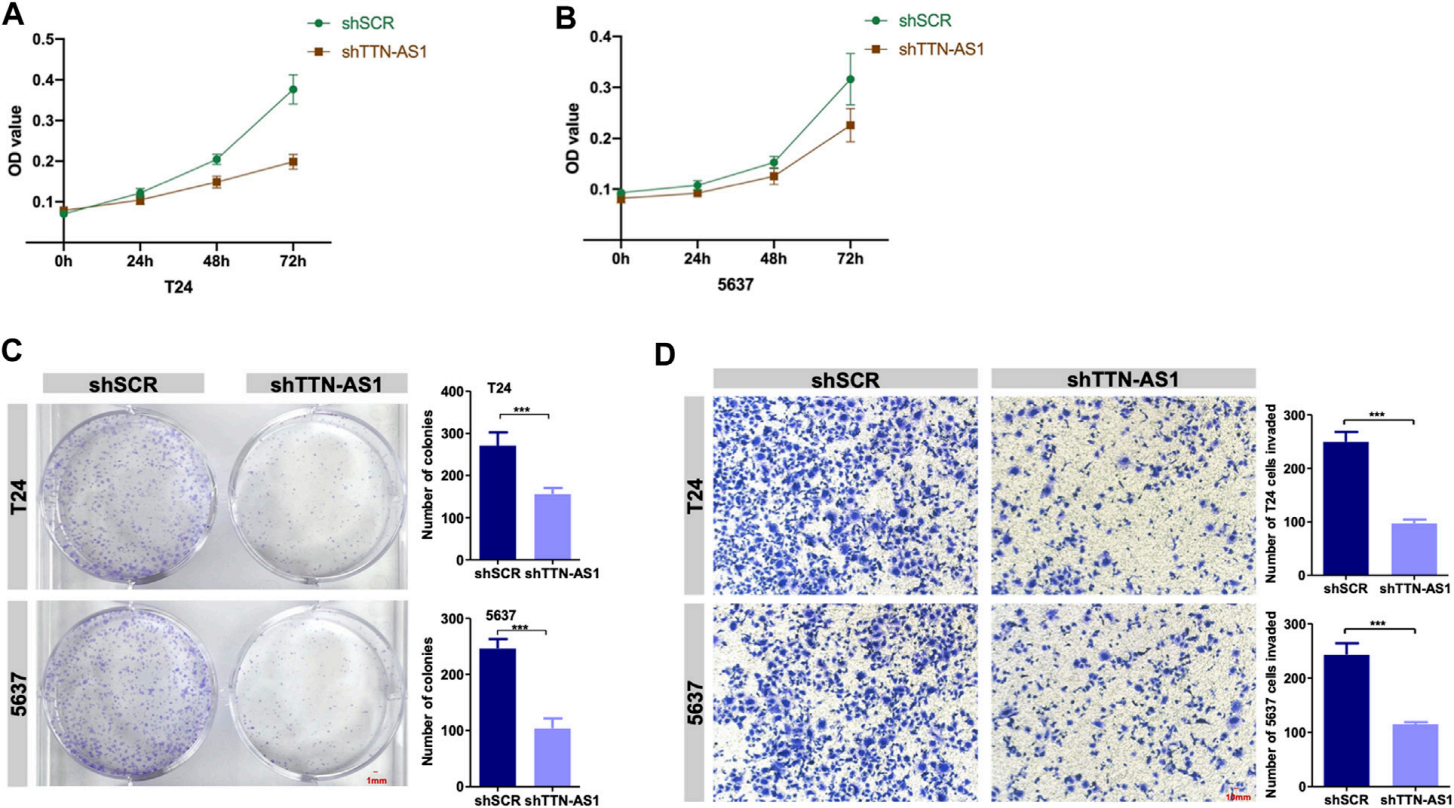
Knockdown of lncRNA TTN-AS1 inhibits the proliferation and invasion of BC cells *in vitro*. **(A,B)** The MTT assay was used to explore the proliferation of two BC cell lines (T24 and 5,637) after the knockdown of lncRNA TTN-AS1. The absorbance value was detected at a wavelength of 490 nm (* means: *p* < 0.05, ** means: *p* < 0.01) **(C)** Colony formation assays was conducted to detected the abilities of proliferation in two BC cell lines (T24 and 5,637) after knocking down lncRNA TTN-AS1 and the number of colonies was counted and plotted on a graph (*** means: *p* < 0.001). **(D)** Transwell invasion assays were conducted to detect the invasiveness of the BC cell lines (T24 and 5,637) after knockdown of lncRNA TTN-AS1, and the number of invaded cells was counted and plotted on a graph (*** means: *p* < 0.001).

To investigate whether lncRNA TTN-AS1 participates in BC growth, a loss of function assay was performed to evaluate T24 and 5,637 cell growth upon TTN-AS1 silencing. Knockdown of TTN-AS1 in T24 and 5,637 cell lines resulted in significant inhibition of cell growth (Figures 2A,B). Consistent with the results in Figures 2A,B, colony assays confirmed the inhibition of cell proliferation in T24 and 5,637 cells after TTN-AS1 knockdown (Figure 2C). Transwell assays demonstrate the significantly decreased abilities of invasion (Figure 2D).

### Targeting lncRNA TTN-AS1 Attenuates BC Growth *in vivo*

**FIGURE 3:**
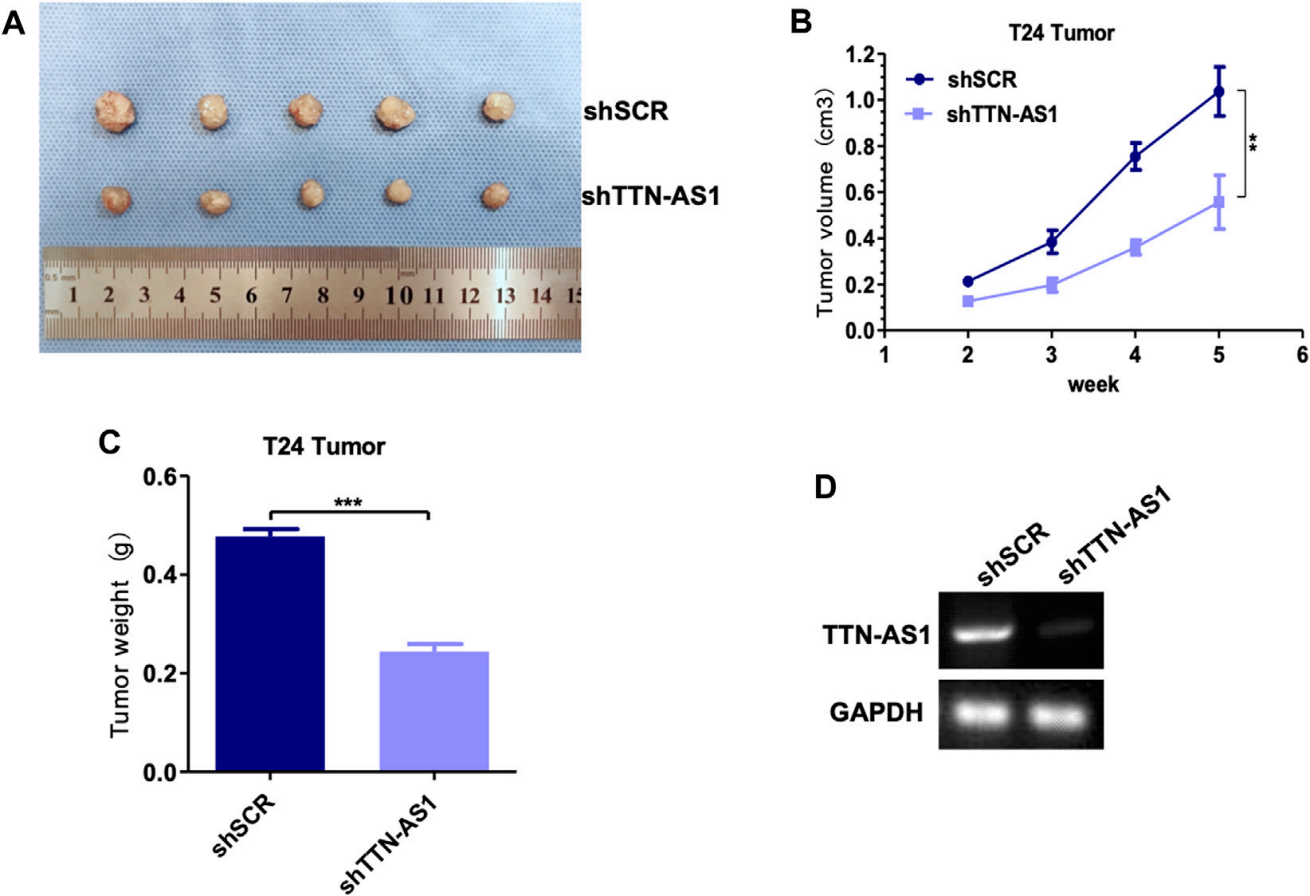
Knocking down lncRNA TTN-AS1 inhibits the growth of bladder tumors *in vivo*. **(A)** The tumor size and morphology were compared between shSCR and shTTN-AS1 groups. **(B)** The tumor volume was analyzed in both shSCR and shTTN-AS1 groups. **(C)** The tumor weight was plotted on a graph (** means: *p* < 0.001). **(D)** qRT-PCR was used to detect the expression level of lncRNA TTN-AS1 at mRNA level from the tumor in both shSCR and shTTN-AS1 groups.

To explore the potential of targeting lncRNA TTN-AS1 in BC, we employed lentiviral injection in T24 tumor xenografts. T24 cells were xenografted into 8-week-old immunocompromised severe combined immunodeficiency (SCID) mice (*n* = 10). The scramble set (shSCR) (*n* = 5) was injected with lentiviruses carrying control scramble shRNA, and the treatment set (shTTN-AS1) (*n* = 5) was injected with lentiviruses carrying lncRNA TTN-AS1 shRNA. Injections were carried out every 3 days for 3 weeks lncRNA TTN-AS1 knocked down during this long-term treatment period led to a significant suppression in the growth of the T24 tumors (Figures 3A–C). The efficiency of silence for TTN-AS1 was validated by PCR in the scramble set and treatment set of tumors (Figure 3D). These results support the potential preclinical significance of targeting lncRNA TTN-AS1 in BC therapy.

### ATF2 Acts as a Downstream Target of lncRNA TTN-AS1 in BC

**FIGURE 4:**
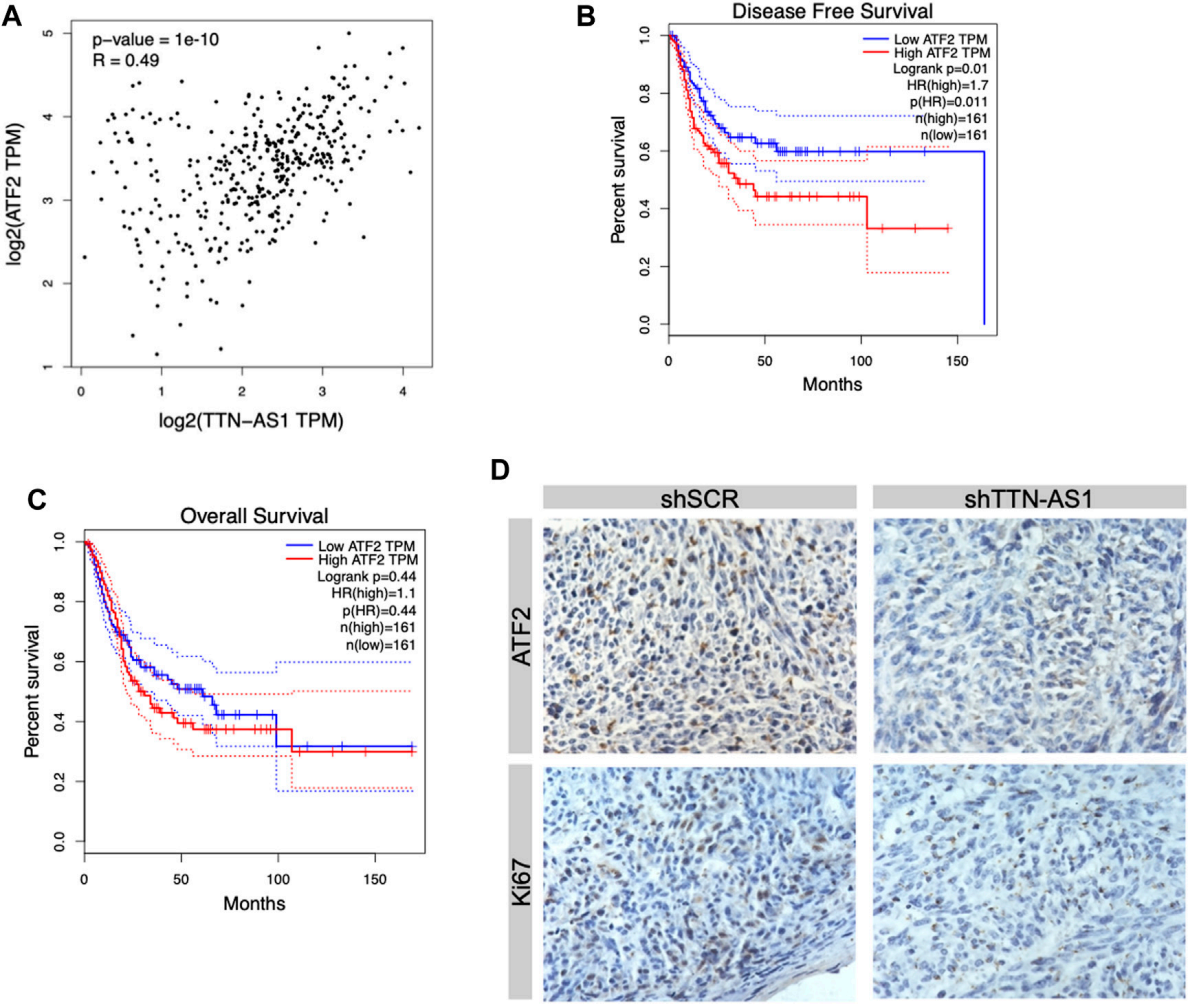
ATF2 was the downstream factor of lncRNA TTN-AS1 and was related to poor prognosis in BC patients. **(A)** Bioinformatics was conducted to analyze the correlation between lncRNA TTN-AS1 and ATF2. **(B)** The DFS of BC patients from TCGA database (*n* = 322, *p* = 0.011) **(C)** The OS of BC patients from TCGA database (*n* = 322, *p* = 0.44) **(D)** IHC was performed to explore the expression of ATF2 in both shSCR and shTTN-AS1 groups.

To explore the downstream of lncRNA TTN-AS1 in BC, we first evaluated its similar expression pattern genes upon the TCGA BC data set by the GEPIA online tool (Tang et al., 2017). ATF2, a member of the AP1 transcription factor family, was listed as the top similar gene of TTN-AS1 (Hai and Curran, 1991; Shaulian and Karin, 2002). A previous study reveals that ATF2 protein is upregulated significantly in BC surgical specimens compared with normal urothelial tissues (Inoue et al., 2018). This study further shows that moderate/strong expression of ATF2 detected by IHC is correlated with poor prognosis of low-grade and muscle-invasive tumors (Inoue et al., 2018). To further investigate the expression relevance of lncRNA TTN-AS1 and ATF2 in BC specimens, we retrieved public TCGA data sets from cBioPortal (Cerami et al., 2012; Gao et al., 2013) and analyzed the two gene-expressed correlation coefficients. We found that these two gene-expressed levels were correlated significantly (Figure 4A). Then, we evaluated ATF2 expression in relation to DFS data as well as OS data on 322 BC patients. We found that BC patients with ATF2 gene high expression had worse DFS (cutoff high expression: 60% of all patients; cutoff low expression: 40% of all patients) (Figure 4B) although its expression has no prediction of OS (Figure 4C). To validate the downstream of lncRNA TTN-AS1, determination of ATF2 protein expression was performed by IHC in xenografts with silence of TTN-AS1 shown in Figure 3A. The results reveal that knockdown of lncRNA TTN-AS1 downregulated the expressed level of ATF2 and ki67 in our *in vivo* T24 tumor xenografts (Figure 4D).

## Discussion

LncRNA, the length of which was more than 200 nt, plays a crucial effect in modulating proliferation, invasion, and migration of various types of tumors, such BC (Li et al., 2020a), renal cancer (Liu et al., 2020), thyoroid cancer (Samimi et al., 2020), and so on. In recent years, more and more studies have found that lncRNA that is abnormally expressed in pan cancer plays an important role in different biological activities, such as autophagy (Cao et al., 2020), epithelial-mesenchymal transition (Wan et al., 2020), cell proliferation (Zhang et al., 2020) and apotosis (Lun et al., 2020). Numerous studies have pointed out that lncRNA plays a vital role in the carcinogenesis, drug resistance, and metastasis of BC. For instance, lncRNA KCNQ1 opposite strand/antisense transcript 1 was found to be overexpressed in BC tissues compared with normal bladder tissues and can promote cell proliferation, migration, invasion, and apoptosis *via* modulating the miR-218–5p/HS3ST3B1 axis (Li et al., 2020a). Except that, Li et al. (2020b), demonstrate that overexpression of insulin-like growth factor binding protein 4-1 could activate the JAK/STAT pathway to promote BC cell proliferation, cell cycle, and cell apotosis. More and more studies have explored that lncRNA TTN-AS1 have participated in carcinogenesis and tumor development and served as a potential therapeutic target in many types of tumors. For example, TTN-AS1 regulates the TTN-AS1/miR-195/cyclin D1 axis through sponge adsorption so as to promote tumor cell proliferation and modulate tumor cell cycle activities in clear cell renal cell carcinoma (Lin et al., 2020). In breast cancer, the abnormally high expression of TTN-AS1 promotes the proliferation, migration, and invasion of tumor cells by inhibiting mir-524 and promoting the expression level of RRM2 (Feng et al., 2020).

So far, no relevant research has pointed out the role of TTN-AS1 in the occurrence and progression of nonmuscular invasive BC. In this study, we found, for the first time, that TTN-AS1 is abnormally highly expressed in BC and is related to the poor prognosis of BLACA patients. In terms of mechanism, TTN-AS1 can promote the proliferation and invasion of tumor cells by regulating the level of downstream factor ATF2. In short, we find that TTN-AS1 can exhibit the role of oncogenes and serve as a therapeutic target for BC through *in vitro* experiments, *in vivo* experiments, and clinical data analysis.

LncRNA regulates tumor mechanisms in a variety of ways, including affecting the transcription of the upstream promoter region of the coding protein gene, mediating chromatin remodeling, histone modification, and changing the cellular localization of proteins, etc., (Chen et al., 2020). ATF2 is reported to play two roles in different types of tumors during tumor progression, namely, tumor-promoting genes and tumor-suppressor factors (Lopez-Bergami et al., 2010). In Inoue’s study, they found that ATF2 acted as an oncogene that promoted cell viability, migration, and invasion of BC *via* the AR signal pathway. Our study analyzes the survival information of BC patients in the TCGA database by bioinformatics and also finds that BC patients in the ATF2 high expression group had adverse DFS. Interestingly, we found that TTN-AS1 and ATF3 were correlated significantly via bioinformatics. Therefore, we propose the hypothesis that ATF2 may be a downstream factor of TTN-AS1 in BC. Furthermore, we used IHC to analyze the protein expression level of ATF2 in tumor tissues in both the knockdown TTN-AS1 and control groups. We find that, after knocking down TTN-AS1, the protein level of ATF2 was also significantly downregulated. Therefore, the oncogenic factor ATF2 of BC may be a potential downstream factor of TTN-AS1. TTN-AS1 promotes the proliferation and invasion of BC cells via inducing the expression of ATF2.

In summary, TTN-AS1 plays a role as a tumor-promoting factor in the progression of BC and can positively regulate the proliferation and invasion of BC cells by regulating the expression of ATF2. At the same time, TTN-AS1 is related to the poor prognosis of BC patients. Therefore, TTN-AS1 can be used as both a potential therapeutic target for Ponge and a predictor to evaluate the prognosis of patients with BC. In addition, this study contains the following shortcoming: the mechanism through which TTN-AS1 regulates the expression of its downstream factor ATF2 requires further experiments to explore.

## Data Availability

The raw data supporting the conclusion of this article will be made available by the authors, without undue reservation.
